# Pentraxin 3 promotes the osteoblastic differentiation of MC3T3-E1 cells through the PI3K/Akt signaling pathway

**DOI:** 10.1042/BSR20201165

**Published:** 2020-06-09

**Authors:** Yong Liu, Hui Wang, Xiao-zhe Zhou, Ning Li, Yi-chao Guo, Tao-ping Chen

**Affiliations:** 1 Department of Orthopedics, The People’s Hospital of Suzhou New District, Suzhou, Jiangsu 215129, China; 2 Department of Orthopedics, The General Hospital of JiZhong Energy Fengfeng Group Co., LTD., 28 North Fuhe Street, Handan 056002, China; 3 Department of Orthopedics, Affiliated Hospital of Hebei University, Baoding 071000, China; 4 Department of Minimally Invasive Spine Surgery, Tianjin Hospital, Tianjin, China; 5 Department of Orthopedics, Baoding Orthopaedic Hospital/People’s Hospital of Lianchi District, Baoding, China

**Keywords:** MC3T3-E1 cells, Osteogenic differentiation, PI3K/Akt, PTX3

## Abstract

Osteoblast cells are responsible for synthesizing new bone tissue, and determining how to control osteoblastic differentiation is vital to the treatment of osteoporosis. In the present study, we show that pentraxin 3 (PTX3) signaling is involved in the regulation of osteoblastic differentiation in MC3T3-E1 cells. Our data reveal that PTX3 is abundantly expressed in MC3T3-E1 cells and that its expression is inducible by the introduction of osteogenic induction medium (OIM). Overexpression of PTX3 was observed to significantly increase the expression of four osteoblast signature genes, including Runt-related transcription factor 2 (RUNX2), alkaline phosphatase (ALP), osteocalcin (OCN) and osterix (OSX), suggesting that the overexpression of PTX3 promotes osteoblastic differentiation. The relative level of gene expression between OIM and OIM plus overexpressed PTX3 was evaluated using the Affymetrix Gene Chip® mouse gene microarray. PTX3-related differentially expressed genes (DEGs) were screened. Gene ontology (GO) functional and Kyoto Encyclopedia of Genes and Genomes database (KEGG) pathway enrichment analyses were performed, and the PI3K/Akt signaling pathway was primarily involved in the osteogenic differentiation of PTX3. Protein–protein interactions (PPIs) were also constructed, and the molecular complex detection (MCODE) plugin calculated modules of PPI networks. Moreover, we show that the effect of PTX3 is mediated by its induction of the PI3K/Akt signaling pathway. Mechanistically, we show that the action of PTX3 requires the activation of PI3K and Akt, and deactivation of PI3K by its inhibitor LY294002 weakens the PTX3-mediated induction of osteoblast signature genes, ALP and matrix mineralization. The present study revealed a new role played by PTX3 and suggest a potential mechanism governing the osteoblastic differentiation of MC3T3-E1 cells.

## Introduction

Osteoporosis is a condition in which bone becomes weak and is characterized by low bone mass and structural deterioration [1,2]. As a result, bone tissue becomes fragile and shows increased vulnerability to fracture. Worldwide, osteoporosis affects approximately 200 million people and is often unrecognized until one encounters the fracture due to the silent nature of the disease [3]. Under healthy conditions, bone is maintained by the constant process of bone remodeling [4].

Normal bone remodeling maintains a balance between bone resorption and formation to maintain bone density. Osteoblasts are generated from the osteogenic differentiation of mesenchymal stem cells (MSCs). MSCs possess the capacity to self-renew and differentiate into multiple cell types. It is known that MSCs are common precursors for osteoblasts [5]. The direction of MSC differentiation depends on specific regulatory factors. RUNX-2 acts as a key transcriptional modulator that mediates the conversion of MSCs into osteoblasts [6,7].

As osteoblasts mature, they begin producing the bone extracellular matrix by secreting bone matrix proteins, including collagen type 1 α 1 (Col-I), osteocalcin (OCN) and alkaline phosphatase (ALP). Type I collagen is the main constituent of bone, comprising the nonmineralized bone matrix. The accumulation of calcium phosphate in the bone matrix by ALP leads to the mineralization of bone. Mature osteoblasts express ALP, Col-I, OCN and osterix (OSX) [4]. It has been recognized that the normal differentiation capacity of MSCs is altered in osteoporosis, thereby hindering osteoblast formation [5].

The PI3K/AKT signaling pathway has been shown to be critical for osteogenic differentiation, growth, and survival [8–10]. PI3K signaling has a positive role in chondrocyte differentiation and is involved in the endochondral bone growth process [11]. Therefore, the PI3K/Akt signaling pathway is crucial for osteoblast differentiation.

Long pentraxin 3 (PTX3), belonging to the pentraxin superfamily, is an inflammatory mediator and plays an essential role in innate immunity and matrix remodeling. PTX3 is induced by primary pro-inflammatory cytokines in different cell types. Mice with a gene deficient in PTX3 show a lower trabecular bone volume, and PTX3 supports maintenance of the bone mass by inhibiting FGF-2 expression [12]. Chang et al. [13] further revealed that PTX3 could stimulate the PI3K/Akt signaling pathway and, in turn, induce metastasis.

Therefore, we investigated whether PTX3 could regulate osteogenic differentiation by regulating the PI3K/Akt signaling pathway in osteoblasts. The present study aimed to investigate the role of PTX3 in promoting osteogenic differentiation in MC3T3-E1 cells. The underlying mechanisms and the connection of the PI3K/Akt signaling pathway with this process were also explored in the present study.

## Materials and methods

### Cell culture

The mouse pre-osteoblast cell line MC3T3-E1 was obtained from American Type Culture Collection (ATCC, CRL-2593). MC3T3-E1 cells were maintained in DMEM (Gibco, U.S.A.) supplemented with 10% FBS (Gibco), 10 mM HEPES (Sigma) and 0.1% penicillin–streptomycin (Sigma–Aldrich).

### Osteogenic differentiation

The differentiation of cultured MC3T3-E1 cells was induced by incubating osteogenic induction medium (OIM) containing DMEM, FBS (5%), β-glycerophosphate (3 mM) and ascorbic acid (50 μg/ml) for 0, 1, 3, 7, 14 and 28 days. Cells maintained in normal growth media were used as the control. After 14 and 28 days of cultivation in an OIM, the ALP activity and Alizarin Red S (ARS) were examined.

### PTX3 overexpression and knockdown assay

The lentiviral expression system overexpressing PTX3 was termed as Lenti-PTX3 OE. The open reading frame of mouse PTX3 (NM_ 602492) was synthesized and cloned into the pL/IRES/GFP plasmid (Novobio, Shanghai, China) to generate pL/IRES/GFP-PTX3. The empty lentiviral expression system without insertion was termed as Lenti-CTRL and used as the control. 293T cells were then transfected with the plasmids listed above. Transfection and lentiviral transduction were performed as described previously [14]. The siRNA sequences against PTX3 and control siRNA was purchased from Thermo Fisher Scientific (Thermo Fisher Scientific, Germany). Experiments with the PTX3 assay were carried out according to the manufacturer’s instructions. Concentration of si-PTX3 was 30 nM and in accordance with the previous studies [15,16].

### Transcriptome sequencing

Total RNA was isolated from MC3T3-E1 cells treated in the presence of OIM with and without PTX3. Following DNA removal by digestion with DNase I (Thermo Fisher Scientific), a single strand of cDNA was first synthesized using random primers, and double-stranded cDNA was subsequently synthesized. The purified double-stranded cDNA was end-repaired, subjected to poly(A) addition and then ligated to the adapter. The cDNA library was then amplified by PCR and subjected to quality checking using an Agilent 2100 Bioanalyzer and ABI StepOnePlus Real-Time PCR System before being sequenced using an Affymetrix Gene Chip® mouse gene microarray.

### Bioinformatics analysis

Gene count normalization and differential expression analysis were performed using the limma and voom package [17]. Following normalization, differentially expressed genes (DEGs) were identified via the R statistical package LIMMA (version 3.5.1) [18]. DEGs with screening conditions of *P*<0.05 and |log2 (fold change)| > 1. Choice of DEGs list using volcano plot and heat map analysis by R software (version 3.5.1).

To explore the function of the DEGs in PTX-3-mediated osteogenic differentiation of MC3T3-E1 cells, the biological function of the DEGs was investigated by a gene ontology (GO) enrichment analysis, which comprised three terms, including biological process (BP), molecular function (MF) and cellular component (CC) [19]. Pathway analysis of DEGs was performed using Kyoto Encyclopedia of Genes and Genomes database (KEGG) pathway analysis. *P*-values <0.05 were considered to be significant. Protein–protein interaction (PPI) analysis between PTX-3 and neighboring genes was performed via the STRING tool (http://string-db.org/), and visualization was constructed by the enrichment map plugin in Cytoscape [20]. Combined scores > 5.0 were selected as previously described [21]. Molecular complex detection (MCODE) plug-in in Cytoscape software (http://www.cytoscape.org/) was performed to screen the modules in the PPI network. The criteria were set as follows: MCODE scores > 3, number of nodes > 3 and *P*-values <0.05 were considered to be significant.

### ALP activity

After reaching full confluence, the cells were equilibrated with ALPL buffer and incubated with 0.2 mg/ml nitro blue tetrazolium and 5-bromo-4-chloro-3-indolyl-phosphate (Sigma, U.S.A.). Cells were stained with ALPL buffer at room temperature for 2 h. The fluorescent signal was measured using a microplate reader (405 nm). The results are expressed as percentage changes in relation to the control group.

### ARS staining

Matrix mineralization was assessed through staining with ARS. After stimulation, the cells were washed followed by fixation with paraformaldehyde (4%) for 10 min. Cells were then stained with ARS (0.2%, pH = 6.8, Solarbio, Beijing, China). Each well was washed three times with distilled water to reduce nonspecific binding. Fluorescence signals were visualized using a fluorescence microscope. Then, ARS was destained for quantitative analyses, as previously described [22]. Briefly, cells were incubated with 0.5 ml of 5% cetylpyridinium chloride. The absorbance was then measured at an OD of 405 nm (Thermo Fisher). Final outcomes were plotted relative to control values.

### RT-PCR and real-time PCR

MC3T3-E1 cells were collected for gene expression profiling related to osteogenic differentiation. To perform qRT-PCR, RNA was isolated using TRIzol (Invitrogen). cDNA was produced from 2 μg RNA with reverse transcriptase, as described by the M-MLV manual (New England Biolabs). The primer sequences used for PCR were as follows: PTX2, forward primer (GCTG GTG CTA GAG GAG CTG) and reverse primer (GGA ATA AAA TAG CTG TTT CAC AAC CT); GAPDH forward primer (GATCGGTACCATCCCAACATCCACAGGTGCTGCTA) and reverse primer (GCAAGAAGCTTTTAAAGATACTTCTCAAG-3). The relative quantity of PTX3 was normalized to GAPDH. The expression of PTX3 was detected using qPCR with SYBR Green Mix Kits (Applied Biosystems). All results were quantified using the 2^−ΔΔ*C*_t_^ relative quantification method.

### Western blot analysis

Whole-cell lysates were made by lysing MC3T3-E1 cells with RIPA buffer. The samples were subjected to 10% SDS/PAGE gel electrophoresis and transferred to a PVDF membrane. The membranes were blocked with blocking solution at room temperature for 2 h on a shaker. Then, PTX3 primary antibody (dilution 1:500, Abcam, ab30734, Cambridge, U.K.), OSX primary antibody (dilution 1:1000, Abcam, ab22552, Cambridge, U.K.), OCN (dilution 1:1000, Abcam, ab13420, Cambridge, U.K.), ALP (dilution 1:1000, Abcam, ab83259, Cambridge, U.K.), Runt-related transcription factor 2 (RUNX2, dilution 1:1000, Abcam, ab192256, Cambridge, U.K.), PI3K (dilution 1:1000, Cell Signaling Technology, #4249, Cambridge, U.K.), p-PI3K (dilution 1:1000, Cell Signaling Technology, #17366, Shanghai, China) and Akt (dilution 1:1000, Cell Signaling Technology, #401 1000, China)   . Then, the membranes were further probed with HRP-conjugated secondary antibody (Abcam, ab6728). After washing three times, the membrane was visualized to determine the activation response using HRP substrates (Thermo Scientific, Rockford, IL, U.S.A.).

### Statistical analysis

The results are presented as the means ± S.D. The multiple group differences were assessed by ANOVA using SPSS software (SPSS software, version 19.0, Munich, Germany). *P*-values <0.05 were considered to be significant.

## Results

### PTX3 is induced for osteoblast differentiation

**Figure 1:**
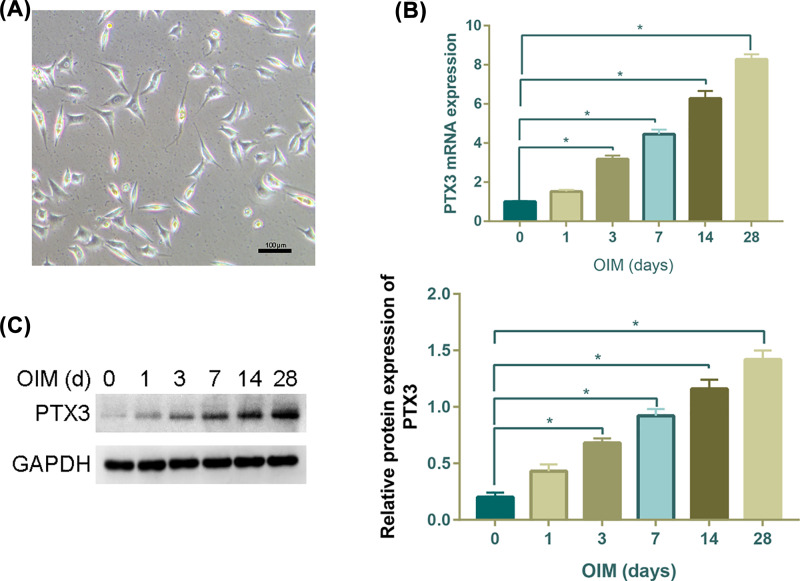
Time-dependent increase in PTX3 osteogenic induction (**A**) Cell morphology of MC3T3-E1 osteoblasts cultivated in culture flasks for 24 h (scale bar  =  100 μm). (**B**) The gene expression of PTX3 was assessed by quantitative real-time PCR after osteogenic induction for 1, 3, 7, 14 and 28 days. (**C**) Western blotting and quantitative analysis using the Western blot assay showed that PTX3 revealed a time-dependent increase in the gray level of osteogenic induction for 1, 3, 7, 14 and 28 days. *P<0.05.

The MC3T3-E1 morphology displayed a spindly fibroblastic shape and was in keeping with previous reports from the literature (Figure 1A). Next, we monitored the expression profile of PTX3 during osteoblastic differentiation of MC3T3-E1 cells. In our experiment, the cells were fed OIM to induce osteoblastic differentiation. Our time-course experiment showed that the mRNA level of PTX3 was gradually induced up to approximately 2.5-, 4-, 6.2- and 8-fold after 3, 7, 14 and 28 days of induction, respectively (Figure 1B).

Meanwhile, the Western blot assay confirmed a similar trend of PTX3 over the extended *in vitro* osteogenic induction time (Figure 1C).

### PTX3 promotes osteoblastic differentiation

The responsive induction of PTX3 suggests its potential role in osteoblastic differentiation. We then treated MC3T3-E1 cells with PTX3 lentivirus while they were fed the osteogenic medium.

**Figure 2:**
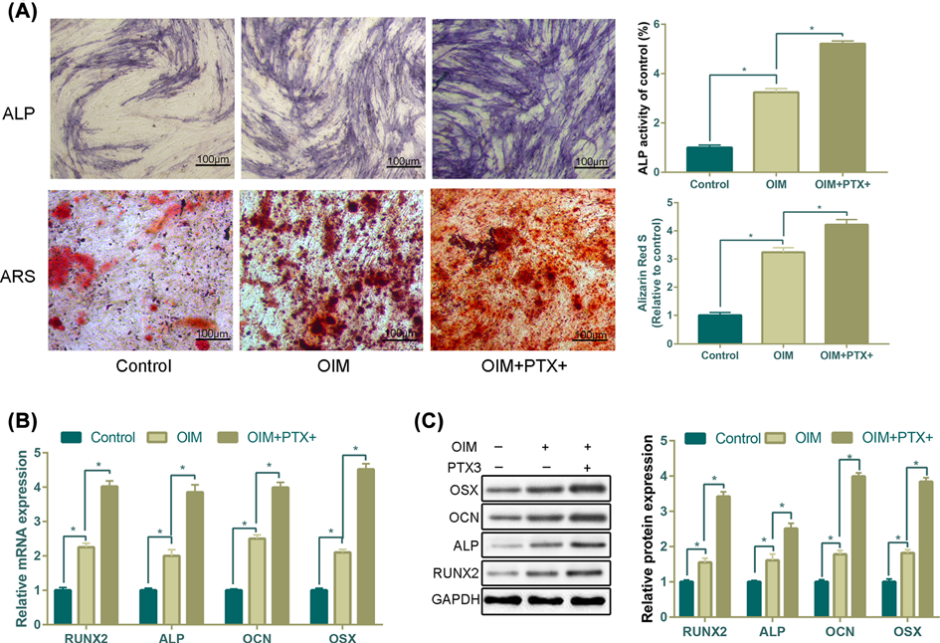
Overexpression of PTX3 promotes osteoblastic differentiation (**A**) ALP staining intensity and ARS staining were enhanced in the PTX3 overexpression group compared with the control group and OIM alone group for 14 and 28 days in the presence or absence of OIM. Cells were cultured in OIM with or without PTX3 for 14 days. Gene levels (**B**) and protein levels (**C**) of RUNX2, ALP, OCN and OSX (*P<0.05).

Osteogenic induction was observed at 14 and 28 days following ALP staining and ARS staining, respectively. Osteogenically induced MC3T3-E1 cells exhibited nearly 2.5- and 3-fold increases in ALP activity and ARS compared with noninduced MC3T3-E1 cells. Additionally, ALP activity and ARS staining were further enhanced following PTX3 overexpression (*P*<0.05, Figure 2A).

We assessed the expression of four osteoblastic signature genes, including RUNX2, ALP, OCN and OSX. Compared with the control group at day 28, the mRNA expression of RUNX2, ALP, OCN and OSX in the MC3T3-E1 cells growing in OIM plus overexpression PTX3 was several-fold higher than that in the MC3T3-E1 cells maintained in OIM alone (Figure 2B).

Moreover, the expression patterns of osteoblastic signature proteins, including RUNX2, ALP, OCN and OSX, detected by WB were consistent with those of mRNA expression (Figure 2C).

### Differentially expressed mRNAs in MC3T3-E1 cells treated with PTX3 for 14 days

**Figure 3:**
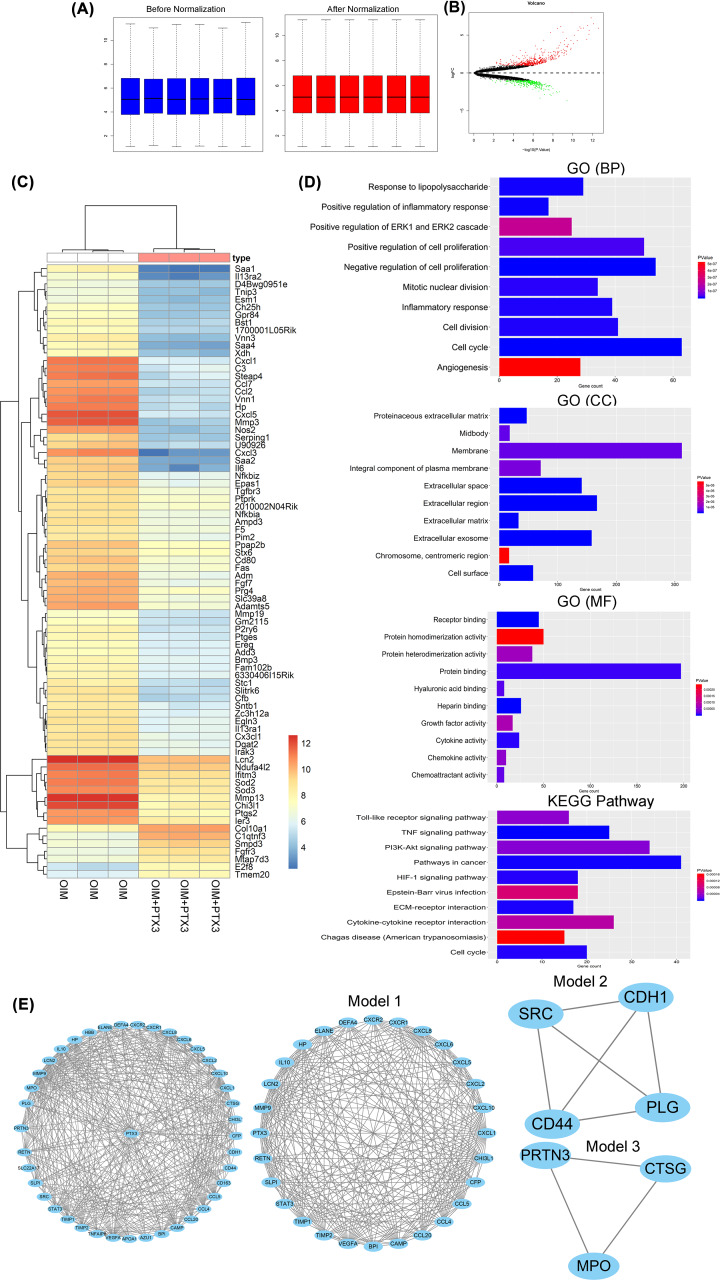
Bioinformatics analysis of the differentially expressed mRNAs in MC3T3-E1 cells treated with PTX3 for 14 days (**A**) Box plots of gene expression data before and after normalization. (**B**) Volcano plot of the DEGs; (red) up-regulated expressed genes; (green) down-regulated genes; and (black) nondifferentially expressed genes. (**C**) Heatmap of the top 80 DEGs (color scheme reflects logarithmic gene expression of each group; highest in red and lowest in blue). (**D**) The results of the GO and KEGG pathway enrichment analyses of the DEGs. (**E**) PPI network, three MCODE models were applied to this network to identify neighborhoods where proteins were densely connected. Three significant modules with a score > 5.0 were selected. Module 1, MCODE score = 6.4, Module 2, MCODE score = 5.2 and Module 1, MCODE score = 5.1.

The mean expression levels of the gene expression profiles are presented in one consistent line after normalization (Figure 3A). The present study investigated six samples, including three PTX3 plus OIM-treated samples and three OIM-alone samples.

A total of 844 differentially expressed mRNAs, including 498 up-regulated and 346 down-regulated mRNAs, were identified between OIM and PTX3 plus OIM (Figure 3B). When we analyzed six MC3T3-E1 cell samples, the 80 top genes clustered into two groups, OIM and OIM plus PTX3, as shown in the heatmap (Figure 3C).

DEG function (GO and KEGG pathway) enrichment analysis revealed several biological pathways that were significantly affected in the up- and down-regulated gene sets. As shown in Figure 3D, GO analysis revealed that 844 DEGs were significantly enriched in the following BPs, including response to lipopolysaccharide, positive regulation of inflammatory response, positive regulation of ERK1 and ERK2 cascade, positive regulation of cell proliferation, negative regulation of cell proliferation, mitotic nuclear division, inflammatory response, cell division, cell cycle and angiogenesis. CC analysis showed proteinaceous extracellular matrix, midbody, membrane, integral component of plasma membrane, extracellular space, extracellular region, extracellular matrix, extracellular exosome, chromosome, centromeric region, and cell surface. In terms of MFs, the DEGs were mainly associated with receptor binding, protein homodimerization activity, protein heterodimerization activity, protein binding, hyaluronic acid binding, heparin binding, growth factor activity, cytokine activity, chemokine activity and chemoattractant activity.

The top ten KEGG pathways that had the most significant enrichment terms were Toll-like receptor signaling pathway, TNF signaling pathway, PI3K/Akt signaling pathway, pathway in cancer, HIF-1 signaling pathway, Epstein–Barr virus infection, ECM–receptor interaction, cytokine–cytokine receptor interaction, Chagas disease (American trypanosomiasis) and cell cycle. We selected the PI3K/Akt signaling pathway for experimental verification.

The PPI network of PTX3 and its neighboring genes consisted of 41 nodes and 159 edges (Figure 3E). There were 28 genes enriched in module 1, 4 genes enriched in module 2 and 3 genes enriched in module 3 when degrees ≥ 5.0 were set as the cut-off criteria.

### PTX3 activates Akt phosphorylation for osteoblastic differentiation

**Figure 4:**
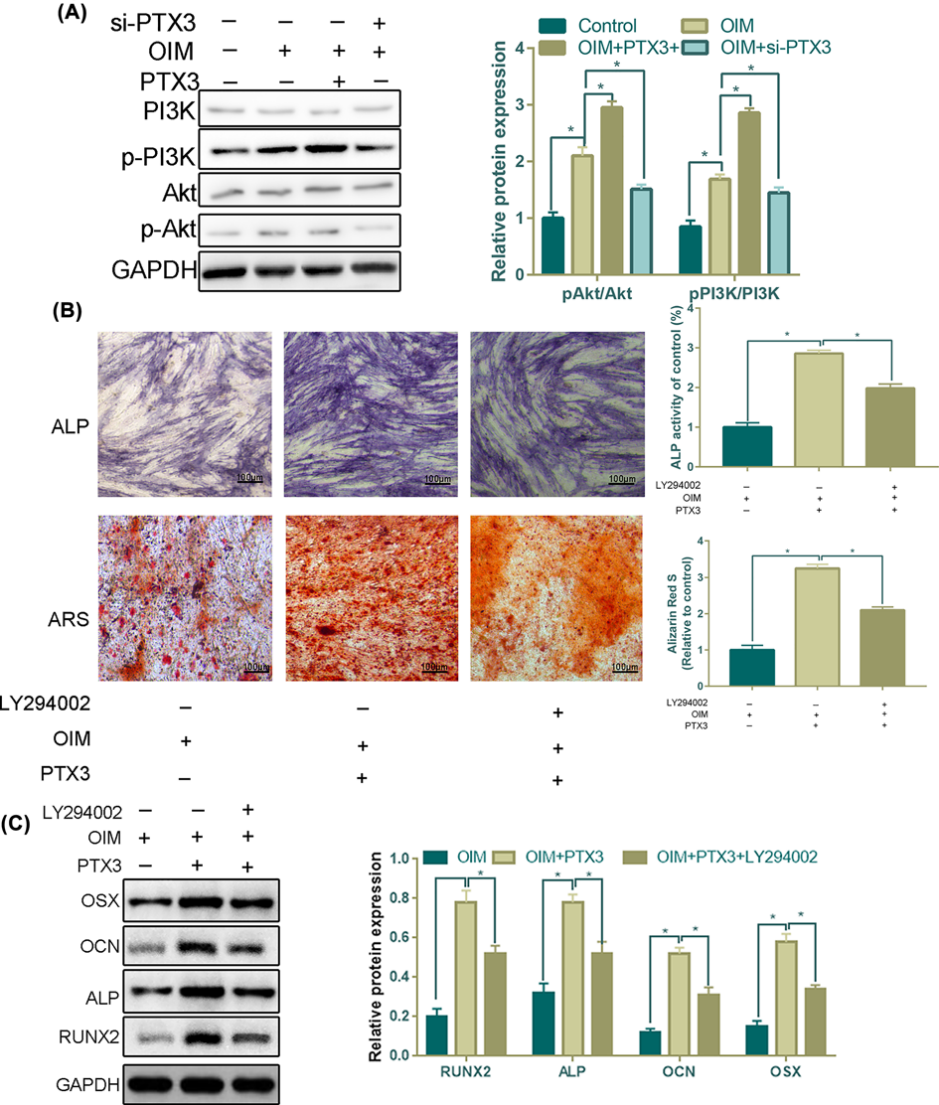
PTX3 participates in the PI3K/AKT signaling pathway (**A**) Western blotting was performed to assess the activation of the PI3K/AKT signaling pathway by detecting PI3K, p-PI3K, AKT and p-AKT in MC33-E1 cells. (**B**) Representative images of ALP staining after 14 days of culture and ARS after 28 days of OIM culture with or without LY294002. (**C**) The PI3K-Akt pathway mediated the elevated effect of PTX3 overexpression during the osteogenic differentiation of MC3T3-E1 cells. MC3T3-E1 cells were incubated with LY294002 for 2 h prior to treatment with PTX3. RUNX2, ALP, OCN and OSX protein expression and quantitative analysis were measured (*P<0.05).

Bioinformatics analysis results found that overexpression of PTX3 mainly activated the PI3K/Akt pathway. Western blot assays suggested that overexpression of PTX3 activated the PI3K/Akt pathway by increasing the phosphorylation of PI3K and Akt (Figure 4A). OIM leads to activation of PI3K and Akt, and an siRNA of PI3K can partially block this activation (Figure 4A).

To verify the involvement of the PI3K/Akt pathway in PTX3-mediated osteoblast differentiation, we added the PI3K-specific inhibitor LY294002 in our 14-day differentiation and 28-day experiment. The results showed that MC3T3-E1 cells in the PTX3 and OIM groups had stronger ARS and ALP staining than those in the OIM group alone (Figure 4B). However, this effect was dramatically attenuated when the cells were pretreated with LY294002, as demonstrated by the ∼1.1-fold increase (Figure 4B).

As an indication of osteoblast differentiation, the expression of osteoblast-associated markers (RUNX2, ALP, OCN and OSX) was examined using a Western blot assay. Compared with the OIM group, the promotion of the effects of PTX3 on the expression of RUNX2, ALP, OCN and OSX was attenuated by pretreatment with LY294002 (Figure 4C). These results indicate that the PI3K/Akt signaling pathway plays an important role in the effects of PTX3 on the osteogenic differentiation of MC3T3-E1 cells.

## Discussion

Osteoblasts are bone-building cells, and the fine regulation of osteogenic differentiation is critical to the process of bone formation, modeling, and remodeling. The understanding of signaling pathways involved in osteogenic differentiation may result in the discovery of novel potential targets of osteoporosis. The most common risk factors for osteoporosis include age, menopause-associated hormone changes in women, changes in physical activity, drugs and other diseases [16,17]. It is widely accepted that age-associated growth hormone, estrogens and other hormones play a key role in the maintenance of bone homeostasis and the development of osteoporosis [18,19].

PTX3 is induced by many cytokines in immune cells and vascular cells and possesses an immune regulatory function [23]. Moreover, PTX3 dysregulation plays an important roles in cancer development [24] and sepsis [25]. A previous study showed that PTX3 expressed precursor osteoblasts and human osteoblast differentiation but not mature osteoblasts [26]. PTX3 is elevated under inflammatory conditions in bone and plays an important role in bone resorption [26].

In the present study, we explored the role of PTX3 in the osteogenic differentiation of MC3T3-E1 cells. Scimeca et al. [27] compared the expression and function of PTX3 by immunohistochemical assay in osteoblasts of osteoporotic, osteoarthritic and young subjects not affected by any bone disorder. The results showed that PTX3 expression was significantly lower in osteoporosis patients than in osteoarthritic and young subjects, which suggested that PTX3 plays a central role in osteoblast proliferation and differentiation. In the present study, we first revealed that the mRNA and protein expression of PTX3 increased with the time of osteogenic differentiation of MC3T3-E1 cells. Our results were different from the study of Lee et al. [26], and PTX3 was also more highly expressed in mature osteoblasts. MC3T3-E1 cells are osteoblast progenitor cells that differ from the multidirectional differentiation potential of stem cells. Then, overexpression of PTX3 by transfection with expression plasmids caused more ALP activity and deposition of calcium salts in the early and late stages, respectively. In particular, both Scimeca et al.’s [28] and Grčević et al.’s [12] groups demonstrated that PTX3 is involved in the deposition of bone matrix.

Further work is necessary to identify the downstream genes of PTX3 and the mechanism of action governing this pathway. Gene chip methods employed samples collected from osteogenic differentiation of MC3T3-E1 cells with or without overexpression of PTX3. A total of 844 DEGs were identified and mainly function in cell proliferation and extracellular space. We further observed strong enrichment of the PI3K/Akt membrane in KEGG enrichment analysis, which was further verified by clinical experiments. It was inferred that the overexpression of PTX3 was associated with a clear increase in the phosphorylation of PI3K and Akt.

The PI3K/Akt signaling pathway plays important roles in the osteogenic differentiation of MC3T3-E1 cells [29]. To further verify whether the promotion effects of PTX3 occurred via Akt, we employed the PI3K inhibitor LY294002. LY294002 abolished the elevated ALP activity and calcium deposition induced by the overexpression of PTX3 when added to MC3T3-E1 cells. Moreover, the inhibition of LY294002 was further confirmed by Western blot analysis for osteoblastic markers. Consistent with our study’s findings, Chang et al. [13] revealed that PTX3 primarily influences the PI3K/Akt signaling pathway in head and neck cancer cell metastasis. Another recent study showed that PTX3 modulated the high-density lipoprotein-induced inflammatory response through the PI3K/Akt signaling pathway [30].

Our study indicates that PTX3 plays important roles in the regulation of osteogenic differentiation of MC3T3-E1 cells. We propose that the activation of PI3K/Akt signaling is a potential mechanism through which PTX3 alters bone metabolism. Further characterization of this newly identified PTX3 for other potential ways and future study using animal models will shed light on its potential therapeutic implications for osteoporosis.

## Abbreviations

ALP: alkaline phosphatase
ARS: Alizarin Red S
BP: biological process
CC: cellular component
Col-I: collagen type 1 α 1
DEG: differentially expressed gene
GAPDH: glyceraldehyde-3-phosphate dehydrogenase
GO: gene ontology
HIF-1: hypoxia
KEGG: Kyoto Encyclopedia of Genes and Genomes database
MCODE: molecular complex detection
MF: molecular function
MSC: mesenchymal stem cell
OCN: osteocalcin
OIM: osteogenic induction medium
OSX: osterix
PPI: protein–protein interaction
PTX3: pentraxin 3
qRT-PCR: quantitative real time polymerase chain reaction
RUNX2: Runt-related transcription factor 2
WB: western blot

## References

[1] Wen J., Guan Z., Yu B., Guo J., Shi Y. and Hu L. (2020) Circular RNA hsa_circ_0076906 competes with OGN for miR-1305 biding site to alleviate the progression of osteoporosis. Int. J. Biochem. Cell Biol. 122, 105719 10.1016/j.biocel.2020.105719 32087327

[2] Zhao Z., Ma X., Ma J., Sun X., Li F. and Lv J. (2018) Naringin enhances endothelial progenitor cell (EPC) proliferation and tube formation capacity through the CXCL12/CXCR4/PI3K/Akt signaling pathway. Chem. Biol. Interact. 286, 45–51 10.1016/j.cbi.2018.03.002 29510123

[3] Li J., Chen X., Lu L. and Yu X. (2020) The relationship between bone marrow adipose tissue and bone metabolism in postmenopausal osteoporosis. Cytokine Growth Factor Rev. 52, 88–98 10.1016/j.cytogfr.2020.02.003 32081538

[4] Sieberath A., Della Bella E., Ferreira A.M., Gentile P., Eglin D. and Dalgarno K. (2020) A comparison of osteoblast and osteoclast in vitro co-culture models and their translation for preclinical drug testing applications. Int. J. Mol. Sci. 21, E912 10.3390/ijms21030912 32019244 PMC7037207

[5] Wang Z. and Bao H.-W. (2019) Cnidium lactone stimulates osteogenic differentiation of bone marrow mesenchymal stem cells via BMP-2/smad-signaling cascades mediated by estrogen receptor. Am. J. Transl. Res. 11, 4984–4991 31497215 PMC6731439

[6] Xu G., Ding Z. and Shi H.-F. (2019) The mechanism of miR-889 regulates osteogenesis in human bone marrow mesenchymal stem cells. J. Orthop. Surg. Res. 14, 366 10.1186/s13018-019-1399-z 31727100 PMC6854696

[7] Gomathi K., Akshaya N., Srinaath N., Moorthi A. and Selvamurugan N. (2020) Regulation of Runx2 by post-translational modifications in osteoblast differentiation. Life Sci. 245, 117389 10.1016/j.lfs.2020.117389 32007573

[8] Mukherjee A. and Rotwein P. (2009) Akt promotes BMP2-mediated osteoblast differentiation and bone development. J. Cell Sci. 122, 716–726 10.1242/jcs.042770 19208758 PMC2720922

[9] Srivastava S., Sharma K., Kumar N. and Roy P. (2014) Bradykinin regulates osteoblast differentiation by Akt/ERK/NFκB signaling axis. J. Cell. Physiol. 229, 2088–2105 10.1002/jcp.24668 24825463

[10] Li H., Yang C., Lan M., Liao X. and Tang Z. (2019) Arctigenin promotes bone formation involving PI3K/Akt/PPARγ signaling pathway. Chem. Biol. Drug Des. 95, 451–459 10.1111/cbdd.13659 31883507

[11] Ulici V., Hoenselaar K.D., Gillespie J.R. and Beier F. (2008) The PI3K pathway regulates endochondral bone growth through control of hypertrophic chondrocyte differentiation. BMC Dev. Biol 8, 40 10.1186/1471-213X-8-40 18405384 PMC2329617

[12] Grčević D., Sironi M., Valentino S., Deban L., Cvija H., Inforzato A. et al. (2018) The long pentraxin 3 plays a role in bone turnover and repair. Front. Immunol. 9, 417 10.3389/fimmu.2018.00417 29556234 PMC5845433

[13] Chang W.-C., Wu S.-L., Huang W.-C., Hsu J.-Y., Chan S.-H., Wang J.-M. et al. (2015) PTX3 gene activation in EGF-induced head and neck cancer cell metastasis. Oncotarget 6, 7741–7757 10.18632/oncotarget.3482 25797258 PMC4480713

[14] Zhou Y.-M., Yang Y.-Y., Jing Y.-X., Yuan T.-J., Sun L.-H., Tao B. et al. (2020) BMP9 reduces bone loss in ovariectomized mice by dual regulation of bone remodeling. J. Bone Miner. Res. 35, 978–993 10.1002/jbmr.3957 31914211

[15] Song T., Wang C., Guo C., Liu Q. and Zheng X. (2018) Pentraxin 3 overexpression accelerated tumor metastasis and indicated poor prognosis in hepatocellular carcinoma via driving epithelial-mesenchymal transition. J. Cancer 9, 2650–2658 10.7150/jca.25188 30087705 PMC6072810

[16] Kim Y., Park J.S., Park H.J., Kim M.K., Kim Y.I., Bae S.K. et al. (2018) Pentraxin 3 modulates the inflammatory response in human dental pulp cells. J. Endod. 44, 1826–1831 10.1016/j.joen.2018.08.003 30477668

[17] Yu K., Chen B., Aran D., Charalel J., Yau C., Wolf D.M. et al. (2019) Comprehensive transcriptomic analysis of cell lines as models of primary tumors across 22 tumor types. Nat. Commun. 10, 3574 10.1038/s41467-019-11415-2 31395879 PMC6687785

[18] Smyth G.K. (2004) Linear models and empirical bayes methods for assessing differential expression in microarray experiments. Stat. Appl. Genet. Mol. Biol. 3, Article3 10.2202/1544-6115.1027 16646809

[19] C. Gene Ontology (2006) The Gene Ontology (GO) project in 2006. Nucleic Acids Res. 34, D322–D326 10.1093/nar/gkj021 16381878 PMC1347384

[20] Merico D., Isserlin R. and Bader G.D. (2011) Visualizing gene-set enrichment results using the Cytoscape plug-in enrichment map. Methods Mol. Biol. 781, 257–277 10.1007/978-1-61779-276-2_12 21877285

[21] Jian L. and Yang G. (2020) Identification of key genes involved in diabetic peripheral neuropathy progression and associated with pancreatic cancer. Diabetes Metab. Syndr. Obes. 13, 463–476 10.2147/DMSO.S235011 32110079 PMC7037175

[22] Shui C. and Scutt A. (2001) Mild heat shock induces proliferation, alkaline phosphatase activity, and mineralization in human bone marrow stromal cells and Mg-63 cells *in vitro*. J. Bone Miner. Res. 16, 731–741 10.1359/jbmr.2001.16.4.731 11316001

[23] Doni A., Garlanda C. and Mantovani A. (2016) Innate immunity, hemostasis and matrix remodeling: PTX3 as a link. Semin. Immunol. 28, 570–577 10.1016/j.smim.2016.10.012 27881292 PMC5414833

[24] Bonavita E., Gentile S., Rubino M., Maina V., Papait R., Kunderfranco P. et al. (2015) PTX3 is an extrinsic oncosuppressor regulating complement-dependent inflammation in cancer. Cell 160, 700–714 10.1016/j.cell.2015.01.004 25679762

[25] Porte R., Davoudian S., Asgari F., Parente R., Mantovani A., Garlanda C. et al. (2019) The long pentraxin PTX3 as a humoral innate immunity functional player and biomarker of infections and sepsis. Front. Immunol. 10, 794 10.3389/fimmu.2019.00794 31031772 PMC6473065

[26] Lee E.-J., Song D.-H., Kim Y.-J., Choi B., Chung Y.-H., Kim S.-M. et al. (2014) PTX3 stimulates osteoclastogenesis by increasing osteoblast RANKL production. J. Cell. Physiol. 229, 1744–1752 10.1002/jcp.24626 24664887

[27] Scimeca M., Salustri A., Bonanno E., Nardozi D., Rao C., Piccirilli E. et al. (2017) Impairment of PTX3 expression in osteoblasts: a key element for osteoporosis. Cell Death Dis. 8, e3125 10.1038/cddis.2017.514 29022895 PMC5682679

[28] Scimeca M., Bonfiglio R., Menichini E., Albonici L., Urbano N., De Caro M.T. et al. (2019) Microcalcifications drive breast cancer occurrence and development by macrophage-mediated epithelial to mesenchymal transition. Int. J. Mol. Sci. 20, 5633 10.3390/ijms20225633 PMC6888678 31718020

[29] Liu M.-Z., Zhou D.-C., Liu Q., Xie F.-L., Xiang D.-X., Tang G.-Y. et al. (2019) Osteogenesis activity of isocoumarin a through the activation of the PI3K-Akt/Erk cascade-activated BMP/RUNX2 signaling pathway. Eur. J. Pharmacol. 858, 172480 10.1016/j.ejphar.2019.172480 31228453

[30] Norata G.D., Marchesi P., Pirillo A., Uboldi P., Chiesa G., Maina V. et al. (2008) Long pentraxin 3, a key component of innate immunity, is modulated by high-density lipoproteins in endothelial cells. Arterioscler. Thromb. Vasc. Biol. 28, 925–931 10.1161/ATVBAHA.107.160606 18218986

